# COVID-19 and sunlight: Impact on SARS-CoV-2 transmissibility, morbidity, and mortality

**DOI:** 10.1016/j.amsu.2021.102419

**Published:** 2021-05-30

**Authors:** Khan Sharun, Ruchi Tiwari, Kuldeep Dhama

**Affiliations:** Division of Surgery, ICAR-Indian Veterinary Research Institute, Izatnagar, Bareilly, 243 122, Uttar Pradesh, India; Department of Veterinary Microbiology and Immunology, College of Veterinary Sciences, Uttar Pradesh Pandit Deen Dayal Upadhyaya Pashu Chikitsa Vigyan Vishwavidyalaya Evam Go Anusandhan Sansthan (DUVASU), Mathura, 281001, India; Division of Pathology, ICAR-Indian Veterinary Research Institute, Izatnagar, Bareilly, 243 122, Uttar Pradesh, India

**Keywords:** COVID-19, SARS-CoV-2, Therapeutics, Photodynamic therapy, Simulated sunlight, Photo-biomodulation

## Abstract

Coronavirus disease 2019 (COVID-19) has already affected millions of people worldwide. There are reports of SARS-CoV-2 transmission as a consequence of environmental contamination. The SARS-CoV-2 laden infective droplets can actively persist on the surface of different materials for several hours to days. Sunlight can affect the stability of SARS-CoV-2 in these aerosols and thereby have an impact on the decay rate of the virus. Solar radiation might play an important role in inactivating SARS-CoV-2 that persists in different surfaces and the environment. Among the different climatological factors, ultraviolet radiation was found to have an important role in determining the spread of SARS-CoV-2. Although ultraviolet radiation C (UVC), UVB, UVA, visible light, and infrared radiation possess germicidal properties, human CoVs including the recently emerged SARS-CoV-2 are inherently sensitive to UVC. However, the successful decontamination using other wavebands requires higher dosages and longer administration times. Furthermore, studies have also identified association between COVID-19 fatalities and the latitude. The intensity of sunlight is highest near the equator, and therefore populations in these regions with more regular exposure to sunlight are less susceptible to vitamin D deficiency. This article has analyzed the potential impact of sunlight in reducing SARS-CoV-2 transmissibility, morbidity, and mortality. It is evident that there exists an interesting link between sunlight exposure, latitude, and vitamin D status with COVID-19 incidence, fatality and recovery rates that requires further investigation.

## Highlights

• Ultraviolet radiation was found to have an important role in determining the spread of SARS-CoV-2.
• Sunlight can affect the stability of SARS-CoV-2 present in the aerosols generated during coughing and sneezing.
• Solar radiation might play an important role in inactivating SARS-CoV-2 that persists in different surfaces and the environment.
• There exists an interesting link between sunlight exposure, latitude, and vitamin D status with COVID-19 incidence, fatality and recovery rates.

## Graphical abstract

**Figure:**
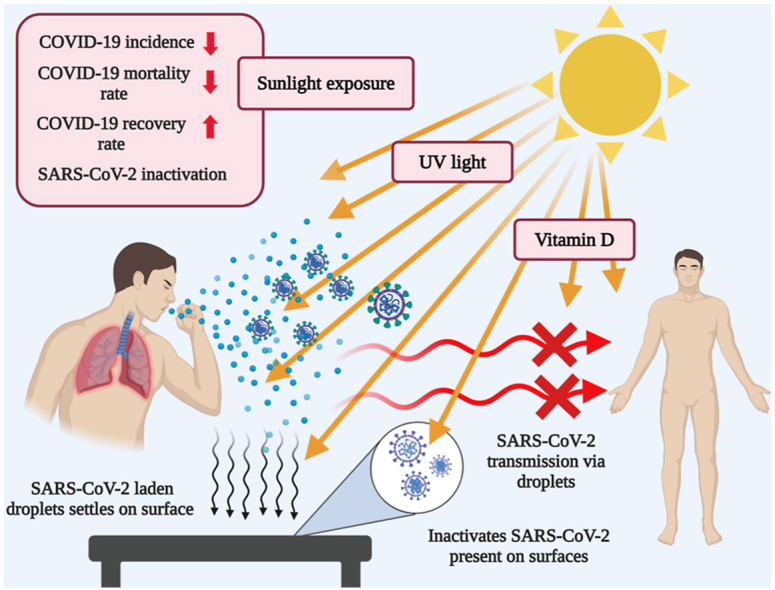

Severe acute respiratory syndrome coronavirus 2 (SARS-CoV-2) was first reported from the Huanan seafood market in Wuhan, Hubei province, China [1]. It has now affected over 164 million people worldwide, with nearly 3.5 million deaths reported globally as of May 18, 2021. SARS-CoV-2 got rapidly spread across the world as a result of the efficient human-to-human transmission. However, the air travel further facilitated the distribution of COVID-19 across international border at a faster rate [2]. SARS-CoV-2 is the sixth coronavirus that can infect human beings [1]. Among the previous coronaviruses (CoVs), SARS-CoV-2 exhibited close genomic similarity with SARS-CoV [3].

High efforts are being made continuously to develop effective vaccines, drugs and therapies to counter this pandemic disease that is posing high global threats and challenges to humanity [[4], [5], [6]]. Photodynamic therapy, photo-biomodulation and light-based technologies have been reported to effectively inactivate SARS-CoV-2 and aid in the management and treatment of COVID-19 during this pandemic crisis [[7], [8], [9], [10], [11]]. The present study aims to analyze the available literature to evaluate the potential role of sunlight in reducing morbidity and mortality associated with SARS-CoV-2 infection in humans. This article also presents an overview of the potential impact of sunlight in reducing SARS-CoV-2 transmissibility.

The ability of infectious particles containing SARS-CoV-2 to persist on different environmental surfaces has contributed to the rapid spread of COVID-19. Therefore, the strategies used to reduce viral diffusion in public environments can help control the ongoing pandemic [12]. Although the evidence suggests possible germicidal properties for UVC, UVB, UVA, visible light, and infrared radiation, available data on UVC are the most robust. The depth of UVC penetration is the lowest among all the wavebands but can deliver adequate virucidal doses in a short duration. However, the successful decontamination using other wavebands requires higher dosages and longer administration times [13]. Human CoVs, including the recently emerged SARS-CoV-2, are inherently sensitive to ultraviolet (UV) radiation [14,15]. A recent in vitro study has confirmed the virucidal activity of UV-C against the clinical isolates of SARS-CoV-2 absorbed on commonly used materials at a working distance of 2–3 cm from the light source [12]. Furthermore, UV-C irradiation was also found to efficiently reduce virus titer (99.99%) on inanimate surfaces such as stainless steel, plastic, and glass at doses ranging from 10.25 to 23.71 mJ/cm^2^ [16].

Sunlight contains a spectrum of UVA, UVB, and UVC, with wave lengths ranging 320–400 nm, 260–320 nm, and 200–260 nm, respectively. Among these, UVC can inactivate CoV, while the synthesis of vitamin D is closely related to UVB radiation exposure [15]. However, both UVA and UVB have poor virucidal activity [17]. Sunlight that reaches the earth's surface contains only UVA and UVB since UVC is absorbed by atmospheric ozone (Fig. 1). Therefore, sunlight reaching the Earth's surface is ineffective in directly eradicating SARS-CoV-2 via virucidal activity [17]. In contrast, Herman et al. (2020) reported that UVB in sunlight can inactivate both SARS-CoV and SARS-CoV-2 present on surfaces as well as in the air. However, inactivation times depend on the latitude, season, and hour of the day [18]. Although the germicidal activity of UVC can be utilized to disinfect personal protective equipment, medical instruments, air, and water, it should not be used in any way that can result in potential exposure to humans [17].

**Fig. 1:**
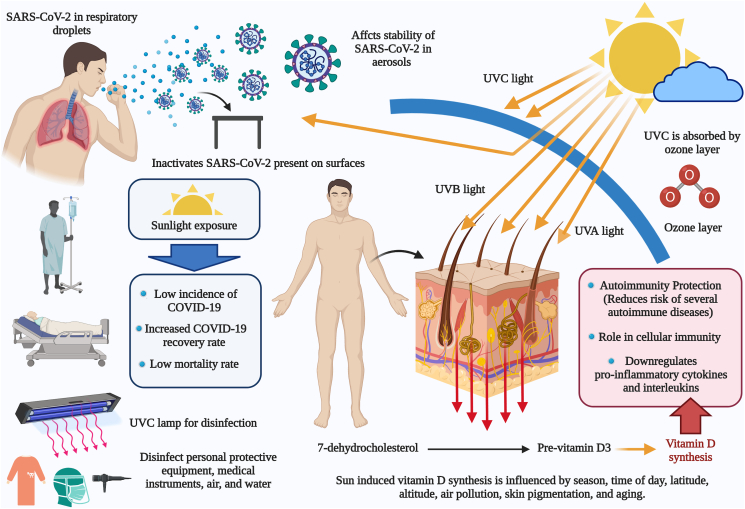
The impact of sunlight exposure in SARS-CoV-2 transmission, morbidity, mortality, and recovery rate.

The relationship between sunlight exposure and the COVID-19 recovery rate was evaluated in Jakarta, Indonesia, by Asyary and Veruswati (2020) [19]. Their findings showed that a higher duration of sunlight exposure was found to be associated with an increase in the recovery rate among patients, indicating the potential of sunlight exposure for accelerating recovery. However, they could not find a correlation between sunlight exposure and the occurrence of COVID-19 and associated death [19]. Direct exposure of skin to sunlight promotes the production of vitamin D, a vital component that regulates the immune system [20]. Vitamin D can lower the risk of respiratory tract infections such as COVID-19 through a multitude of cellular interactions that involves reduction in the production of inflammatory cytokines, maintaining endothelial integrity, and increasing ACE2 concentrations [21].

The intensity of sunlight is highest near the equator, and therefore populations in these regions with more regular exposure to sunlight are less susceptible to vitamin D deficiency. High COVID-19 associated deaths in northern US states are hypothesized to be the result of vitamin D deficiency in African-Americans [22]. If the link between disease severity and vitamin D status is found causative, COVID-19 could become seasonal [23]. The consumption of adequate vitamin D might limit the spread of SARS-CoV-2 especially in individuals with dark skin tone, excess body fat, and genetic predisposition to vitamin D deficiency [22]. Therefore, vitamin D supplementation can be done among the susceptible population with darker skin especially during the dark months of winter and spring season [22,23]. Although high dose vitamin D consumption (>4000 IU/day) should be recommended only after performing clinical trials, supplementation at moderate doses can be suggested for individuals at risk of deficiency [23].

Furthermore, studies have identified a link between COVID-19 fatality rate and latitude, further confirming the relationship between sunlight exposure and COVID-19 mortality [20]. The link between latitude and COVID-19 mortality was further confirmed based on the data obtained from USA [22]. Therefore, the countries that are closer to the equator had comparatively lower fatality rates as compared to the countries that are farther away [20,22,23]. Nakada and Urban (2020) studied the different environmental factors that could have influenced the spread of SARS-CoV-2 in São Paulo, Brazil. The results suggest that COVID-19 infection rate is inversely correlated with UV radiation and temperature, indicating a possible role for sunlight in decreasing SARS-CoV-2 infectivity [24]. In a descriptive observational cross-sectional study conducted in France with a sample set of 64, 553, 275 individuals, a significant negative correlation was observed between sunlight exposure and COVID-19 mortality rate [25]. In another ecological study conducted in the different regions of Italy, the received solar UV radiation was found to have an impact on the incidence of COVID-19 and the occurrence of disease complications [26]. However, the statistical outcomes may not confirm the existence of a specific cause-effect relationship between received solar UV radiation and the disease variables such as incidence and mortality rate.

Among the different climatological factors, ultraviolet radiation was found to have an important role in determining the spread of COVID-19 [27]. Higher incidence of COVID-19 was linked to increased population density and reduced solar irradiance [27]. SARS-CoV-2 was found to be highly susceptible to irradiation with UVC light. The viral stock with a high infectious titer (5 × 10^6^ TCID_50_/mL) got completely inactivated within 9 min of exposure. Complete inactivation was achieved with a UVC dose of 1048 mJ/cm^2^ [14]. However, complete inactivation was not achieved while the virus was exposed to UVA light indicating weak inactivation potential.

There are reports of SARS-CoV-2 transmission as a consequence of environmental contamination. SARS-CoV-2 can actively persist on the surface of different materials for several hours to days [28,29]. SARS-CoV-2 aerosolized from the infected patients could remain infective over the surface for considerable amount of time especially during the winter further increasing the risk for re-aerosolization and subsequent infections. Available data also indicate that SARS-CoV-2 can get inactivated relatively easily as compared to influenza A during summer [29]. Solar radiation might play an important role in inactivating SARS-CoV-2 that persist in different surfaces and the environment [27]. Therefore, sunlight might have a role in deciding the occurrence, spread, and duration of COVID-19 pandemics [29].

Similarly, available evidence suggests that sunlight UV radiation dose is negatively correlated with the percentage of patients testing positive for human coronaviruses (CoVHKU1, CoVOC43, CoVNL63, and CoV229E), including SARS-CoV-2 in the United States [15]. In another ecological study (multiple-group design), ultraviolet radiation was found to be significantly related to the incidence of COVID-19 and disease severity (based on hospital and ICU admissions). In addition, temperature was found to be the main climatic factor responsible for the difference in SARS-CoV-2 spread across Spanish regions [30]. Carleton et al. (2021) evaluated the combined spatially resolved dataset from 3235 regions across 173 countries to analyze the relationship between COVID-19 cases and local environmental conditions. The findings indicate that UV exposure can decrease the COVID-19 growth rate [31]. Similarly, countries present in the lower temperature regions were found to be associated with a rapid increase in the COVID-19 cases compared to the countries in warmer climatic regions [32]. The temperature may play a major role in viral transmissibility as it has a direct impact on virus viability and host immunity. However, the relationship between temperature and SARS-CoV-2 transmissibility is complex in nature [33]. The hot air generated in enclosed spaces such as parked vehicles as a result of solar heating is a promising strategy for disinfection since SARS-CoV-2 is inactivated at 56 °C (within 30 min) [34]. However, passive solar heating is a sustainable technique with benefits of no added costs that can be used only in countries with hot climate.

SARS-CoV-2 can get rapidly transmitted through the respiratory droplets generated during sneezing or coughing. Sunlight can affect the stability of SARS-CoV-2 present in these aerosols and thereby have an impact on the decay rate of the virus [11]. A study that evaluated the impact of relative humidity and sunlight on the stability of aerosolized SARS-CoV-2 identified sunlight as an important factor that can influence SARS-CoV-2 transmission via aerosols [11]. Furthermore, sunlight can rapidly inactivate SARS-CoV-2 present on the surfaces, thereby affecting its persistence on surfaces and subsequent exposure risk [9]. It can be hypothesized that natural sunlight may act as an effective disinfectant for contaminated nonporous materials. Antimicrobial photodynamic therapy can be considered as an alternative therapeutic strategy against SARS-CoV-2 and involves the use of safe and cost-effective photosensitizers (phenothiazines or porphyrins). This technique can be used to develop photoactive fabrics that can disinfect surfaces, air and wastewater both under natural sunlight and artificial light [35].

Therefore, based on the available data, the incidence, mortality, and recovery rate in patients with COVID-19 is considered to be correlated with sunlight exposure and vitamin D levels. We can also hypothesize that there exists an interesting link between sunlight exposure, latitude, and vitamin D status with COVID-19 incidence, fatality and recovery rates. However, further studies are warranted to estimate the optimum level of sunlight exposure as well as the possible role of vitamin D supplementation for decreasing the incidence and fatality rate of COVID-19 in high-risk populations.

## Ethical approval

Not applicable.

## Source of funding

No substantial funding to be stated.

## Author contribution

All authors contributed equally - study concept or design, data collection, data analysis or interpretation, writing the paper.

## Research registration number

1. Name of the registry: Not applicable.

2. Unique Identifying number or registration ID: Not applicable.

3. Hyperlink to your specific registration (must be publicly accessible and will be checked): Not applicable.

## Guarantor

**Dr. Khan Sharun**, Division of Surgery, ICAR-Indian Veterinary Research Institute, Izatnagar, Bareilly-243 122, Uttar Pradesh, India. Email: sharunkhansk@gmail.com.

**Dr. Kuldeep Dhama**, Division of Pathology, ICAR-Indian Veterinary Research Institute, Izatnagar, Bareilly-243 122, Uttar Pradesh, India. Email: kdhama@rediffmail.com.

## Provenance and peer review

Not Commissioned, internally reviewed.

## Consent

Not applicable.

## Declaration of competing interest

All authors declare that there exist no commercial or financial relationships that could, in any way, lead to a potential conflict of interest.

## References

[1] Dhama K., Khan S., Tiwari R., Sircar S., Bhat S., Malik Y.S., Singh K.P., Chaicumpa W., Bonilla-Aldana D.K., Rodriguez-Morales A.J. (2020). Coronavirus disease 2019-COVID-19. Clin. Microbiol. Rev..

[2] Sharun K., Tiwari R., Natesan S., Yatoo M.I., Malik Y.S., Dhama K. (2020). International travel during the COVID-19 pandemic: implications and risks associated with 'Travel Bubbles'. J. Trav. Med..

[3] Sharun K., Sircar S., Malik Y.S., Singh R.K., Dhama K. (2020). How close is SARS-CoV-2 to canine and feline coronaviruses?. J. Small Anim. Pract..

[4] Rabaan A.A., Al-Ahmed S.H., Sah R., Tiwari R., Yatoo M.I., Patel S.K., Pathak M., Malik Y.S., Dhama K., Singh K.P., Bonilla-Aldana D.K., Haque S., Martinez-Pulgarin D.F., Rodriguez-Morales A.J., Leblebicioglu H. (2020). SARS-CoV-2/COVID-19 and advances in developing potential therapeutics and vaccines to counter this emerging pandemic. Ann. Clin. Microbiol. Antimicrob..

[5] Sharun K., Tiwari R., Iqbal Yatoo M., Patel S.K., Natesan S., Dhama J., Malik Y.S., Harapan H., Singh R.K., Dhama K. (2020). Antibody-based immunotherapeutics and use of convalescent plasma to counter COVID-19: advances and prospects. Expet Opin. Biol. Ther..

[6] Vellingiri B., Jayaramayya K., Iyer M., Narayanasamy A., Govindasamy V., Giridharan B., Ganesan S., Venugopal A., Venkatesan D., Ganesan H., Rajagopalan K., Rahman P.K.S.M., Cho S.G., Kumar N.S., Subramaniam M.D. (2020). COVID-19: a promising cure for the global panic. Sci. Total Environ..

[7] Enwemeka C.S., Baker T.L., Greiner J.V., Bumah V.V., Masson-Meyers D.S., Castel J.C., Vesonder M. (2020). Antimicrobial photodynamic therapy as a potential treatment against COVID-19: a case for blue light. Photobiomodul Photomed Laser Surg.

[8] Fernandes A.B., de Lima C.J., Villaverde A.G.J.B., Pereira P.C., Carvalho H.C., Zângaro R.A. (2020). Photobiomodulation: shining light on COVID-19. Photobiomodul Photomed Laser Surg.

[9] Ratnesar-Shumate S., Williams G., Green B., Krause M., Holland B., Wood S., Bohannon J., Boydston J., Freeburger D., Hooper I., Beck K., Yeager J., Altamura L.A., Biryukov J., Yolitz J., Schuit M., Wahl V., Hevey M., Dabisch P. (2020). Simulated sunlight rapidly inactivates SARS-CoV-2 on surfaces. J. Infect. Dis..

[10] Sabino C.P., Ball A.R., Baptista M.S., Dai T., Hamblin M.R., Ribeiro M.S., Santos A.L., Sellera F.P., Tegos G.P., Wainwright M. (2020). Light-based technologies for management of COVID-19 pandemic crisis. J. Photochem. Photobiol., B.

[11] Schuit M., Ratnesar-Shumate S., Yolitz J., Williams G., Weaver W., Green B., Miller D., Krause M., Beck K., Wood S., Holland B., Bohannon J., Freeburger D., Hooper I., Biryukov J., Altamura L.A., Wahl V., Hevey M., Dabisch P. (2020). Airborne SARS-CoV-2 is rapidly inactivated by simulated sunlight. J. Infect. Dis..

[12] Criscuolo E., Diotti R.A., Ferrarese R., Alippi C., Viscardi G., Signorelli C., Mancini N., Clementi M., Clementi N. (2021). Fast inactivation of SARS-CoV-2 by UV-C and ozone exposure on different materials. Emerg. Microb. Infect..

[13] Horton L., Torres A.E., Narla S., Lyons A.B., Kohli I., Gelfand J.M., Ozog D.M., Hamzavi I.H., Lim H.W. (2020). Spectrum of virucidal activity from ultraviolet to infrared radiation. Photochem. Photobiol. Sci..

[14] Heilingloh C.S., Aufderhorst U.W., Schipper L., Dittmer U., Witzke O., Yang D., Zheng X., Sutter K., Trilling M., Alt M., Steinmann E., Krawczyk A. (2020). Susceptibility of SARS-CoV-2 to UV irradiation. Am. J. Infect. Contr..

[15] Tang L., Liu M., Ren B., Wu Z., Yu X., Peng C., Tian J. (2021). Sunlight ultraviolet radiation dose is negatively correlated with the percent positive of SARS-CoV-2 and four other common human coronaviruses in the U.S. Sci. Total Environ..

[16] Gidari A., Sabbatini S., Bastianelli S., Pierucci S., Busti C., Bartolini D., Stabile A.M., Monari C., Galli F., Rende M., Cruciani G., Francisci D. (2021). SARS-CoV-2 survival on surfaces and the effect of UV-C light. Viruses.

[17] O'Connor C., Courtney C., Murphy M. (2020). Shedding light on the myths of ultraviolet radiation in the COVID-19 pandemic. Clin. Exp. Dermatol..

[18] Herman J., Biegel B., Huang L. (2020). Inactivation times from 290 to 315 nm UVB in sunlight for SARS coronaviruses CoV and CoV-2 using OMI satellite data for the sunlit Earth. Air Qual Atmos Health.

[19] Asyary A., Veruswati M. (2020). Sunlight exposure increased Covid-19 recovery rates: a study in the central pandemic area of Indonesia. Sci. Total Environ..

[20] Whittemore P.B. (2020). COVID-19 fatalities, latitude, sunlight, and vitamin D. Am. J. Infect. Contr..

[21] Mercola J., Grant W.B., Wagner C.L. (2020). Evidence regarding vitamin D and risk of COVID-19 and its severity. Nutrients.

[22] Kohlmeier M. (2020). Avoidance of vitamin D deficiency to slow the COVID-19 pandemic. BMJ Nutr Prev Health.

[23] Rhodes J., Dunstan F., Laird E., Subramanian S., Kenny R.A. (2020). COVID-19 mortality increases with northerly latitude after adjustment for age suggesting a link with ultraviolet and vitamin D. BMJ Nutr Prev Health.

[24] Nakada L.Y.K., Urban R.C. (2020). COVID-19 pandemic: environmental and social factors influencing the spread of SARS-CoV-2 in São Paulo, Brazil. Environ. Sci. Pollut. Res. Int..

[25] Lansiaux É., Pébaÿ P.P., Picard J.L., Forget J. (2020). Covid-19 and vit-d: disease mortality negatively correlates with sunlight exposure. Spat Spatiotemporal Epidemiol.

[26] Isaia G., Diémoz H., Maluta F., Fountoulakis I., Ceccon D., di Sarra A., Facta S., Fedele F., Lorenzetto G., Siani A.M., Isaia G. (2020). Does solar ultraviolet radiation play a role in COVID-19 infection and deaths? An environmental ecological study in Italy. Sci. Total Environ..

[27] Guasp M., Laredo C., Urra X. (2020). Higher solar irradiance is associated with a lower incidence of coronavirus disease 2019. Clin. Infect. Dis..

[28] Li Q., Guan X., Wu P., Wang X., Zhou L., Tong Y., Ren R., Leung K.S.M., Lau E.H.Y., Wong J.Y., Xing X., Xiang N., Wu Y., Li C., Chen Q., Li D., Liu T., Zhao J., Liu M., Tu W., Chen C., Jin L., Yang R., Wang Q., Zhou S., Wang R., Liu H., Luo Y., Liu Y., Shao G., Li H., Tao Z., Yang Y., Deng Z., Liu B., Ma Z., Zhang Y., Shi G., Lam T.T.Y., Wu J.T., Gao G.F., Cowling B.J., Yang B., Leung G.M., Feng Z. (2020). Early transmission dynamics in Wuhan, China, of novel coronavirus-infected pneumonia. N. Engl. J. Med..

[29] Sagripanti J.L., Lytle C.D. (2020). Estimated inactivation of coronaviruses by solar radiation with special reference to COVID-19. Photochem. Photobiol..

[30] Cacho P.M., Hernández J.L., López-Hoyos M., Martínez-Taboada V.M. (2020). Can climatic factors explain the differences in COVID-19 incidence and severity across the Spanish regions?: an ecological study. Environ. Health.

[31] Carleton T., Cornetet J., Huybers P., Meng K.C., Proctor J. (2021). Global evidence for ultraviolet radiation decreasing COVID-19 growth rates. Proc. Natl. Acad. Sci. U. S. A..

[32] Iqbal M.M., Abid I., Hussain S., Shahzad N., Waqas M.S., Iqbal M.J. (2020). The effects of regional climatic condition on the spread of COVID-19 at global scale. Sci. Total Environ..

[33] Ran J., Zhao S., Han L., Liao G., Wang K., Wang M.H., He D. (2020). A re-analysis in exploring the association between temperature and COVID-19 transmissibility: an ecological study with 154 Chinese cities. Eur. Respir. J..

[34] Wang X., Sun S., Zhang B., Han J. (2020). Solar heating to inactivate thermal-sensitive pathogenic microorganisms in vehicles: application to COVID-19. Environ. Chem. Lett..

[35] Almeida A., Faustino M.A.F., Neves M.G.P.M.S. (2020). Antimicrobial photodynamic therapy in the control of COVID-19. Antibiotics (Basel).

